# Localization of the questionnaire about sharps disposal at home among diabetes based on knowledge, attitude, and practice theory, and a cross-sectional survey of current conditions

**DOI:** 10.3389/fpubh.2024.1355510

**Published:** 2024-05-28

**Authors:** Haijing Zan, Tao Liu, Zhixing Meng, Jingru Wang

**Affiliations:** School of Nursing, Jinzhou Medical University, Jinzhou, Liaoning, China

**Keywords:** diabetes mellitus, sharps, disposal, patients, knowledge-attitude-practice model

## Abstract

**Background:**

Diabetes Mellitus is a long duration disease, and if a person with diabetes is infected with a blood-borne infectious disease and proper syringe disposal practices are not followed, they run the danger of transmitting the infection to others for a very long period. Whereas fewer research has been done in China on the handing of sharp objects at home. Therefore, there is a need to translate and localize the Knowledge-Attitude-Practice Questionnaire regarding sharp disposal for diabetic patients to assess the current level of patient knowledge, attitudes, and practices and to improve the basis for promoting safe sharps handling practices.

**Methods:**

This investigation was a cross-sectional study. The Knowledge-Attitude-Practice Questionnaire regarding sharp disposal was localized and debugged and tested for reliability and validity, and then 334 patients were investigated by General Characteristics Questionnaire, Knowledge- Attitude-Practice Questionnaire regarding sharp disposal, and the influencing factors of practice level regarding sharp disposal of patients were analyzed.

**Results:**

The Cronbach’s α value of the attitude section was 0.864 and the content validity index was 0.923. The knowledge and practice sections are in line with continental language conventions and are easy to understand without any ambiguity. The majority (52%) of the participants had poor knowledge and a neutral attitude toward disposing of sharp objects. Sharps disposal practices among diabetes mellitus patients were poor since about 90% of patients dispose of their used sharps directly into the household waste. Furthermore, we found that level of education, knowledge and attitude were the major predictors of practices regarding sharps disposal among diabetic patients (*R*^2^ = 0.573, *p* < 0.001).

**Conclusion:**

The Chinese version of the Knowledge-Attitude-Practice Questionnaire regarding sharp disposal has applicability in China. In China, current practice of disposing used sharps is improper. Additionally, the majority of the subjects had low levels of knowledge and attitudes. To raise awareness and encourage diabetic patients to follow appropriate sharps disposal practices, there needs to be ongoing education and a locally tailored safe sharp disposal alternative accessible.

## 1 Introduction

Diabetes is now a major public health concern on a global scale. The International Diabetes Federation (IDF) estimates that 536.6 million people are living with diabetes in 2021, and this number is projected to increase by 46%, reaching 783.2 million by 2045. It was reported that China had 141 million diabetes in 2021, making it the country with the biggest diabetic population worldwide (1). The volume of sharps-related medical waste connected with diabetes will probably grow due to the diabetes epidemic. Sharps are medical equipment with sharp points or edges that can cut or pierce flesh, stated the World Health Organization (WHO) (2). Insulin pen needles, insulin syringes, lancets, reusable insulin pens, disposable insulin pens, insulin vials or cartridges, and insulin pump devices are some of the sharps frequently used for home treatment in the case of diabetes patients (3). Self-monitoring of blood glucose is frequently required for patients to maintain good glycemic control. Most patients also require daily insulin injections, which have been recognized as the foundation of diabetes care due to their therapeutic benefits in glycemic control (4). According to the WHO, almost 8 billion of the over 16 billion injections given each year occur outside of medical facilities (5). If not properly disposed of, these sharp objects constitute a threat to the general populace including patients themselves, kids, other family members, and people in the general community and raise the possibility of the spread of blood-borne illnesses (6). A case of improper use of insulin pens was reported in the United States, resulting in 16 people contracting hepatitis C, again demonstrating the potential for transmission of blood-borne diseases through insulin injections (7).

In many locations, sharp things are appropriately disposed of in hospitals, but this is not the situation with the general population (8–10). A study in Pakistan showed that more than 90% of patients in the community dispose of used sharps directly into domestic waste (11). The same study conducted in Peninsular Malaysia also found that 75% of the subjects incorrectly handled the sharps they used (12). China is no exception, many studies on reducing the incidence of needlestick injuries and the safe handling of needles have been conducted in hospitals (13–15), while few studies on community disposal have been reported.

In some developed countries, in response to this problem, the relevant authorities have tried a number of approaches with varying degrees of success. A study in Guyana had considerable success in collecting used insulin syringes in exchange for free new syringes by providing empty tablet containers to diabetic patients at primary clinics (6). Diabetes UK’s recommendation to use opaque hard plastic containers to store used sharps was practiced in the majority of the subjects (16).

This is a matter of concern, especially in many developing countries where waste disposal systems are limited and there are no comprehensive laws and regulations (17), such as in India (18). In addition, healthcare professionals frequently concentrate on education on how to correctly administer insulin or check blood sugar levels, but frequently neglect the need of safe handling of sharp items (19). Therefore, most patients were unaware that sharps from injections that needed to be disposed of properly. According to Kotchen et al. (20), patients were three times more likely to bring their waste to the designated collection center for final disposal when they were informed of the risks associated with incorrect disposal. In a survey of 250 diabetic patients, Tarik et al. found that 67% of patients had never been told how to properly dispose of used needles or pens (21). Studies have indicated that patients who are aware of how to obtain pertinent health information are more likely to handle sharps safely. Since medical personnel are involved in all facets of diabetes care, it is their duty to impart their expertise and confidence to patients (22). In China, sharps waste in community settings has not yet received much attention. The medium-sized city of Jinzhou faces the same issue as the nation as a whole. Although a previous study was conducted in a southern Chinese city (23), the conclusions of this study are not necessarily applicable to the Jinzhou population due to the different economic, cultural and geographic contexts. Furthermore, in contrast to earlier research, this study uses the Knowledge, Attitude, Practice theoretical framework to thoroughly examine the influencing elements behind the region’s present sharps disposal practices.

Therefore, the aim of this study was to introduce a relevant foreign questionnaire in order to investigate the current status of diabetic patients’ knowledge, attitudes, and practice regarding sharps disposal and to explore the factors influencing them. These indicators will serve as a foundation for researchers and local health care policy makers to create public health intervention plans that are tailored to Jinzhou’s diabetic population.

## 2 Materials and methods

### 2.1 Study design

This was a cross-sectional study using convenience sampling to explore the prevalence of and factors associated with the knowledge, attitude and practice of used insulin needles among Chinese patients with diabetes.

### 2.2 Participants

A cross-sectional study was conducted from May to September 2023 at two hospitals in Jinzhou City with 334 participants. Patients who had been diagnosed with diabetes for at least 6 months and were using a home glucometer or insulin, as well as insulin injected by the patient himself were included in the study. Patients using insulin pumps, females diagnosed with gestational diabetes and inability to communicate with the researcher were excluded. Before testing, the respondent is fully told of the survey’s relevance and aim, and their informed consent is gained by having them sign an informed consent form.

### 2.3 Measures

#### 2.3.1 Demographic characteristic

General Characteristics Questionnaire included age, sex, education level, marital status, professional situation, living area, monthly income, family history of diabetes, disease duration and insulin administration time, etc.

#### 2.3.2 Knowledge-attitude-practice questionnaire

The questionnaire was developed by Choo et al. (12). With the consent of this author, researchers following the Brislin model to translate KAP Questionnaire into Chinese (24). The English version of the scale was independently translated by two nursing graduate students who were proficient in English and had passed the CET-6. Reverse translation was carried out independently by two nursing experts who were fluent in English and had no interaction with the original scale. To get to a consensus, the two post-translated scales were compared, and the variations between the two translations were spoken about. In this study, five researchers, including an infectious diseases doctor, an endocrinologist and three nursing specialists, formed an expert panel. In order to bring the language expressions more in line with continental language conventions, the panel compared the translation and back versions with the original scale while accounting for cultural adaptation and conceptual equivalency of idioms. In the end, convenience sampling was used to choose 30 diabetic individuals for the pilot study. Patients reported that the subject matter was clear and that they had no trouble understanding the semantics.

The KAP is a 28-item, three-parts questionnaire, aims to measure the situation of knowledge, attitudes, and practices of diabetic patients regarding sharps disposal. The knowledge section consisted of 10 questions and 4 options with only one best answer, and the sections assessed the proper use of sharps, the dangers of improperly disposing of sharps, and the proper way to handle sharps. One point is awarded for a correct answer to a question, up to a maximum of 10 points. Among them, the total score of this part is divided into three grades: good (score ≥ 8), medium (score 6–7), poor (score < 6).

The attitude section consisted of 9 items and the Likert 5-level scoring method was adopted. The scale ranging from 5 (strongly agree) to 1 (strongly disagree), and for negative statements, a reverse scale was used. Subjects were asked to select one answer for each entry and the maximum score was used to identify the different attitudes. In this study, the overall Cronbach’s α scale was 0.864, which has good reliability.

The practice section consisted of 9 items used to elicit information about the subjects’ procedures for disposing of sharp objects. The subjects covered in the practice segment included where to dispose of insulin needles and needle containers, where to dispose of insulin needles when one uses a sharp outside one’s home, whether or not needles are reused, history of receiving advices regarding sharp disposal, etc.

### 2.4 Ethical considerations

The study was approved by the center’s ethics committee before it began (Ethics Approval Number: JZMULL2023071).

### 2.5 Data collection

The questionnaire was distributed in person at the location by the researcher. Patients who satisfied the inclusion and exclusion criteria were given instructions on the purpose, prerequisites, and manner of completion before signing an informed consent form. After being completed, the anonymously completed surveys were immediately gathered. Upon completion, the surveys were immediately collected, and patients were notified of any missing information. According to Kendall’s sample calculation method, samples were measured to be 5–10 times the number of scale items with the most items (25). The Knowledge-Attitude-Practice Questionnaire consists of 28 items, taking into account a 20% sample loss. As a result, the calculated sample size was at least 168–336. A total of 350 questionnaires with missing data were distributed in this study, and 334 valid questionnaires were recovered, with an effective rate of 95%, meeting the sample size requirements.

### 2.6 Statistical analysis

All analyses were conducted using SPSS 25. The study population was described using mean ± standard deviation (Mean ± SD) and *n* (%). The relationships between sociodemographic factors and knowledge, attitude, and practice scores of diabetic patients were expressed by *T*-test or one-way ANOVA analyses. Variables with the value of *p* < 0.05 were accepted as candidates for multivariate linear regression analysis to identify the independent factor associated with the sharp disposal practices of patients. Pearson Correlation was used to measure the relationship between knowledge, attitude and practice.

## 3 Results

### 3.1 Validity and reliability analysis

The Kaiser-Meyer-Olkin (KMO) test was 0.923, and Bartlett sphericity test was significant (χ^2^ = 1308.167; *p* < 0.001). A panel of five experts evaluated the translated scale for content validity, the I-CVI was 0.80 to 1.00, and the S-CVI was 0.827. The Cronbach’s α value of the attitude section was 0.864.

### 3.2 Baseline characteristics of the participants

Table 1 describes the demographics of diabetic. Among the 334 patients, 40.1% of them were in the age groups of 60–70 years. Over half of the respondents was the man (58.7%), and almost all (83.5%) of the patients reported being married. In addition, 80.8% of the patients lived in urban areas and the educational level of the participants was junior or senior high school (62%). There were 82 patients (24.6%) in an employed situation and nearly half of the participants had an average monthly income of around 3,000 RMB. Regarding the disease related information, 46.4% of patients with a disease course of 10–20 years and 173 patients (51.8%) with injections for more than 5 years. Moreover, more than half of patients needed twice injections a day. In this study, most of them had no family history of diabetes (64.4%). The factors associated with knowledge and attitude of diabetic are shown in Table 1.

**Table 1:** Characteristics of diabetic patients that related to knowledge and attitude (*n* = 334).

Variables	Overall sample *N* (%)	Knowledge			Attitude		
		Mean ± SD	*t* or *F*	*p* value	Mean ± SD	*t* or *F*	*p* value
Sex							
Male	196 (58.7)	5.49 ± 2.06	2.117	0.130	29.35 ± 4.46	1.160	0.125
Female	138 (41.3)	5.02 ± 1.88			28.80 ± 3.82		
Age (years)							
<60	103 (30.8)	6.24 ± 1.59	40.974	<0.001	30.38 ± 3.55	30.869	<0.001
60–70	134 (40.1)	5.51 ± 1.86			30.04 ± 4.13		
>70	97 (29.0)	3.99 ± 1.91			26.53 ± 3.86		
Level of education							
Primary and below	91 (27.2)	3.69 ± 1.54	68.533	<0.001	25.95 ± 3.15	53.122	<0.001
Junior or senior high school	207 (62.0)	5.68 ± 1.79			29.96 ± 3.92		
College and above	36 (10.8)	7.17 ± 1.42			32.33 ± 3.41		
Marital status							
Married	279 (83.5)	5.50 ± 1.92	4.259	0.373	29.53 ± 4.19	4.114	0.126
Single/divorced/widowed	55 (16.5)	4.27 ± 2.09			27.04 ± 3.67		
Professional situation							
Unemployed/retired	252 (75.4)	4.95 ± 2.01	−5.846	0.002	28.71 ± 4.33	−3.211	0.004
Employed	82 (24.6)	6.37 ± 1.53			30.40 ± 3.56		
Living area							
Rural	64 (19.2)	4.14 ± 1.70	−5.352	0.108	26.95 ± 3.26	−4.728	0.005
City	270 (80.8)	5.57 ± 1.97			29.64 ± 4.25		
Monthly income (RMB)							
<3,000	189 (56.6)	4.61 ± 1.88	30.378	<0.001	27.96 ± 3.96	18.651	<0.001
3,000–5,000	124 (37.1)	6.12 ± 1.82			30.55 ± 4.19		
>5,000	21 (6.3)	6.57 ± 1.66			31.19 ± 3.23		
Family history of diabetes							
Present	119 (35.6)	5.34 ± 1.90	0.327	0.200	29.24 ± 4.29	0.390	0.814
Absent	215 (64.4)	5.27 ± 2.06			29.06 ± 4.18		
Duration of diabetes (years)							
<10	132 (39.5)	5.56 ± 2.01	3.042	0.049	29.45 ± 4.05	1.008	0.366
10–20	155 (46.4)	5.24 ± 1.99			29.04 ± 4.46		
>20	47 (14.1)	4.74 ± 1.92			28.47 ± 3.79		
Duration of insulin use (years)							
≤5	161 (48.2)	5.52 ± 2.05	1.195	0.097	29.53 ± 4.18	1.727	0.900
>5	173 (51.8)	5.09 ± 1.93			28.74 ± 4.22		
Schedule of daily insulin injections							
Once	29 (8.7)	5.48 ± 1.96	0.648	0.524	29.69 ± 4.56	0.541	0.583
Twice	174 (52.1)	5.18 ± 1.99			28.92 ± 4.19		
Three times or more	131 (39.2)	5.41 ± 2.02			29.27 ± 4.18		

### 3.3 Level of patients with knowledge, attitude, and practice

Table 2 provides a summary of diabetic patients’ attitudes on disposal of sharp objects. 51.8% of the individuals had a neutral attitude toward disposing of sharp objects, compared to 37.4% of the subjects who had a favorable view.

**Table 2:** Levels of attitude regarding sharps disposal among diabetic patients (*n* = 334).

Attitude level	Total number of subjects (334)
Strongly Negative	6 (1.8%)
Negative	30 (9.0%)
Neutral	173 (51.8%)
Positive	125 (37.4%)
Strongly Positive	0 (0%)

Table 3 depicts the degree of knowledge about sharp disposals among subjects. The average knowledge scores among subjects for disposing of sharp objects were 5.30 ± 2.00. A total of 115 subjects had a good knowledge level, 45 subjects had a moderate knowledge level. In contrast, 174 subjects had a poor knowledge level regarding sharp disposal.

**Table 3:** Level of knowledge regarding sharps disposal among diabetic patients (*n* = 334).

Knowledge level	Total number of subjects (334)
Good	115 (34%)
Moderate	45 (14%)
Poor	174 (52%)

Table 4 provided an overview of the subjects’ sharps disposal practices.

**Table 4:** Sharps disposal practices among diabetic patients (*n* = 334).

Variables	Frequency (%)
Disposal practice when they at home	
Discard into a rubbish bin	304 (91.0%)
Discard into a special container	30 (9%)
others	0
Disposal practice when they inject outside	
Discard into a rubbish bin	319 (95.5%)
Put in the special container and bring it back to their house	11 (3.3%)
others	4 (1.2%)
History of reusing insulin needles	
No	23 (6.9%)
Yes	311 (93.1%)
History of reusing lancet	
No	284 (85%)
Yes	50 (15%)
Previous instruction on sharps disposal	
No	280 (83.8%)
Yes	54 (16.2%)
Accidentally stabbed with a needle yourself	
No	288 (86.2%)
Yes	46 (13.8%)
Family members have been accidentally stabbed with a needle	
No	317 (94.9%)
Yes	17 (5.1%)
Re-cap needle with the cover after using	
No	97 (29%)
Yes	237 (71%)
Receiving any diabetes training or briefing	
No	184 (55.1%)
Yes	150 (44.9%)

### 3.4 Correlation between knowledge, attitude, and practice in diabetic

The average score of knowledge, attitude, and practice of diabetic and their correlation are shown in Table 5. Correlation analysis showed that there was a positive correlation between knowledge and attitude (r = 0.782, *p* < 0.001), knowledge was positively correlated with practice (r = 0.682, *p* < 0.001), attitude was positively correlated practice (r = 0.727, *p* < 0.001).

**Table 5:** Correlation between knowledge, attitude and practice in diabetic patients (*n* = 334).

Variables	Mean ± SD	Knowledge r (*p*-value)	Attitude r (*p*-value)
Knowledge	5.30 ± 2.00	1	
Attitude	29.12 ± 4.21	0.782**	1
Practice	13.17 ± 1.61	0.682**	0.727**

** *p* < 0.01.

### 3.5 Predictors of practice in diabetic

A study of crucial variables influencing diabetics’ practice levels. The total score of practice in diabetic was set as a dependent variable, the variables that were statistically significant in the univariate analysis were subjected to a multifactorial analysis, and the results of the analysis showed that the level of education, knowledge, and attitude had a significant effect on the practice in diabetes, as shown in Table 6.

**Table 6:** Multiple linear regression analysis of sharps disposal practice among diabetic patients (*n* = 334).

Variables	B	SE	β	*t*	*p*
(Constant)	6.873	0.686		10.012	<0.001
Age	−0.102	0.109	−0.049	−0.934	0.351
Level of education	0.355	0.139	0.131	2.550	0.011
Professional situation	−0.378	0.193	−0.101	−1.957	0.051
Monthly income	0.129	0.124	0.049	1.040	0.299
Knowledge	0.196	0.050	0.244	3.942	<0.001
Attitude	0.175	0.022	0.458	7.772	<0.001

## 4 Discussion

The results suggest that the questionnaire is a valid and reliable measurement tool for measuring the current status of home sharps disposal among Chinese diabetic patients. This cross-sectional study used this questionnaire to examines the level of knowledge, attitude, and practice regarding sharp disposals among patients with Diabetes Mellitus in Jinzhou, China and explores factors associated with sharps disposal practices. In this study, the data depicted that almost half of the patients had poor knowledge regarding sharp disposal, around 52%. This found was similar to many studies: Egypt (19), Ethiopia (26), South Africa (27), and Malaysia (17). This might be explained by the fact that in many countries, the increasing prevalence of diabetes has caused an imbalance in healthcare, with the majority of medical professionals continuing to emphasize safe injections in their training (18). However, it is equally important to overlook the safe disposal of waste. Although some of the patients in this study claimed that they did not receive relevant information in the hospital, but rather through family and friends and the media, doctors and nurses are still the mainstay of health education in China. Therefore, we advise healthcare organizations to do a better job of educating individuals about the safe handling of sharp objects, especially in elementary institutions. In our study, half of the patients (51.8%) had a neutral level of attitude toward sharps disposal, which was lower than the attitude level reported by studies conducted in Malaysia (12) and Egypt (19). This shows that they do not care about learning how to properly handle sharps and the responsibility of causing a needlestick injury. This also suggests that we should focus on the dangers of randomly discarding sharp objects in health education to raise patients’ awareness of this issue ideologically.

This study found significant differences in the level of knowledge and attitude about sharps disposal among diabetic patients with different demographic characteristics. Level of education is an influential factor in the knowledge, attitude score of diabetic patients regarding sharps disposal. The scores for knowledge and attitude increase with education level. Patients with a high level of education have been educated for a long time, have a wide range of knowledge, have many ways of acquiring knowledge, have a strong sense of initiative, and have a more positive attitude toward acquiring health (26). This serves as a reminder for healthcare professionals to concentrate on patients with poor education level and convey the information in simple terms to make sure the patient understands. The finding that patients with high monthly incomes had better knowledge and attitudes about sharps disposal, which was similar with other study (28). Patients with lower incomes tend to satisfy their basic needs for food and clothing, and most of their requests for insulin-related injection behavior are to alleviate the pain caused by the disease, and they do not devote too much time and energy to waste disposal, so their attitudes are on the negative side.

Age affects the people’s memory and comprehension, making it harder for older adults to learn new things on their own, while at the same time having less access to knowledge than younger people. In the present study, patients with diabetes under 60 years old scored higher on knowledge and attitude tests. As far as professional situation is concerned, the results of this study showed that employed patients were more informed and positive attitude. However, the results of the multiple regression showed that employed patients had poorer practice behaviors. One plausible explanation is that employed patients need to cope with the social and workplace pressures of the workplace, and the safe disposal of sharps associated with insulin injections can add to the burden of day-to-day disease management, especially when injections are administered in the workplace (29). As a result, even though they have more comprehensive knowledge and more information, they are poorer in terms of practice. In addition, living area also influences the patient’s attitude regarding sharps disposal. The reason for this may be that patients living in rural areas were often accompanied by a low level of literacy (30).

According to our results, about 90% of patients dispose of their used sharps directly into the household waste. There was a low prevalence of safe sharps disposal practices. This finding is similar to previous study conducted in other countries (19, 26, 31), but higher than Saudi Arabia (8) and Sri Lanka (10). In China, diabetes patients do not have access to sharps container disposal and instead dispose of them in home waste because there are no sharps collection terminals or community sharps disposal initiatives. At the same time, 93% of patients had reused insulin needles, some respondents said they were unaware that needles were not reusable, and some claimed that they did so for financial reasons. In our study, 83% of subjects claimed that they had not received instruction on safe sharps handling in the past, this finding supports research on another study (32). It is therefore important for healthcare providers to educate people with diabetes about their health and help them to safely dispose of used sharps and develop safe disposal behaviors through a nationally structured health education module that incorporates viable options. Regression analysis showed that level of education, knowledge and attitude were the major predictors of practices regarding sharps disposal among diabetic. Better understanding and mastery of pertinent information brought about by literacy in turn raises awareness of the significance of safe disposal, rectifies negative attitudes, and produces positive results (33). Some studies have shown that people with diabetes who possess awareness of the risk of transmission of bloodborne pathogens are more likely to take safety measures (23).

The theory of knowledge, attitude, and practice (KAP) points that by acquiring knowledge about health and building positive attitudes, individuals can eventually develop healthy behaviors (34). This study found a positive correlation between knowledge, attitude, and practices regarding sharps disposal. This implies that the diabetic patient’s behavioral compliance will be better the more knowledgeable he or she was about properly disposing of sharps and the more positive attitude about doing so. In order to achieve the best results from safety education, healthcare providers should continue to assess patients’ knowledge, attitude, and behavior during future interventions. They should also quickly correct any misconceptions or bad habits that patients may have.

However, while there is a recursive relationship between knowledge, attitude, and practice, there is no necessary relationship (35). In this study, knowledge and attitude levels were average, but practice levels were poor, showing that having some information and a healthy attitude are not necessarily translated into good practices. Due to financial restrictions on healthcare or other considerations, there is a gap between information, attitudes, and safe behaviors. Therefore, facilitating the implementation of sharps disposal related to diabetic patients can be done at a higher level of change, such as the development of appropriate measures by the state.

### 4.1 Limitations

This study has some limitations. First, almost all of the diabetic patients in this study had type 2 diabetes, type 1 diabetes is less common and more of this type can be included in future studies. Second, it’s possible that some individuals had trouble correctly recalling earlier occurrences, such as needle stick injuries, therefore, the possibility of recall bias cannot be ruled out. Third, convenience sampling was used in this study to draw participants from one region, limiting the generalizability of our findings. There is a need for a multi-center investigation with bigger samples. Interventional studies may also be taken into account.

## 5 Conclusion

The theory of knowledge, attitude, and practice is being used for the first time in China in this study to the practice of disposing of sharps in diabetic patients’ homes. The study showed that the prevalence of insulin-related sharps disposal in the diabetic population is low in China. Improper disposal of sharps into the community and surrounding environment is a significant public health concern. A multi-pronged strategy may be needed to address these issues, such as comprehensive training for healthcare professionals or more extensive health promotion initiatives, in addition to providing advice on safe handling in the instructions for injecting devices. The findings of this study increase the need for reflection by National Health System staff. We suggest that primary care services can be considered the best place for an intervention study, as the majority of patients are managed for their condition in primary hospitals.

## Data availability statement

The raw data supporting the conclusions of this article will be made available by the authors, without undue reservation.

## Ethics statement

The studies involving humans were approved by this study was following the principles of the Declaration of Helsinki and was approved by the Jinzhou Medical University Ethics Committee (Approval No. JZMULL2023071). All study participants received informed consent. The studies were conducted in accordance with the local legislation and institutional requirements. The participants provided their written informed consent to participate in this study. Written informed consent was obtained from the individual(s) for the publication of any potentially identifiable images or data included in this article.

## Author contributions

HZ: Writing – original draft, Writing – review & editing. TL: Writing – review & editing. ZM: Writing – review & editing. JW: Writing – review & editing.
